# Evidence for non-conservative current-induced forces in the breaking of Au and Pt atomic chains

**DOI:** 10.3762/bjnano.6.241

**Published:** 2015-12-09

**Authors:** Carlos Sabater, Carlos Untiedt, Jan M van Ruitenbeek

**Affiliations:** 1 Huygens–Kamerlingh Onnes Laboratory, Leiden Institute of Physics, PO Box 9504, 2300 RA Leiden, Netherlands; 2 Departamento de Física Aplicada, Universidad de Alicante, Campus de San Vicente del Raspeig, E-03690 Alicante, Spain

**Keywords:** atomic chain, atomic-size contacts, break junctions, current-induced forces

## Abstract

This experimental work aims at probing current-induced forces at the atomic scale. Specifically it addresses predictions in recent work regarding the appearance of run-away modes as a result of a combined effect of the non-conservative wind force and a ‘Berry force’. The systems we consider here are atomic chains of Au and Pt atoms, for which we investigate the distribution of break down voltage values. We observe two distinct modes of breaking for Au atomic chains. The breaking at high voltage appears to behave as expected for regular break down by thermal excitation due to Joule heating. However, there is a low-voltage breaking mode that has characteristics expected for the mechanism of current-induced forces. Although a full comparison would require more detailed information on the individual atomic configurations, the systems we consider are very similar to those considered in recent model calculations and the comparison between experiment and theory is very encouraging for the interpretation we propose.

## Introduction

One of the great promises of nanotechnology is the prospect of constructing autonomous nanomachines. While this is still a far prospect, micromotors [1] and nanomotors [2–3] have already been demonstrated. A drawback of these nanomotors is that they require externally supplied cyclic driving. It would be very attractive if we could realize a nanomotor driven by a dc current flow, in analogy to the driving of a waterwheel by a waterflow. For this to work one would need a non-conservative force to act between the non-equilibrium electron bath and the position degrees of freedom of the ions that make up the nanoscale system.

Such a non-conservative force mechanism was recently proposed in works by Dundas et al. [4–5], and these ideas were further elaborated upon by Lü et al. [6] and by Bode et al. [7]. In these works the electron gas is treated fully quantum-mechanically, while the lattice degrees of freedom are described in a semi-classical approximation. The interaction between the electrons and the lattice gives rise to two interesting contributions to the force on the ions, which appear only when the electron gas is driven out of equilibrium, such that a macroscopic current flows through the system. The first is a non-conservative force, in other words a force that can do work on the ions, and which is linked to the wind force often discussed in the context of electromigration [8]. The second is a force originating in the Berry phase of the electrons, which works just like a Lorentz force: it is directed perpendicular to the velocity of the ions, keeping them in periodic orbits.

This so-called ‘Berry force’ has never been demonstrated in experiment. In fact, also the first component of the non-equilibrium force is only known from electromigration experiments that detect mass transport of atoms or ions. Truly microscopic experiments probing the forces at the scale of atoms or molecules have not yet been reported. Such experiments are challenging, because high current densities are required and it would probably be needed that the detailed atomic configurations are known and can be monitored. A first promising experiment that fulfils many of these conditions was recently reported [9].

A concrete model system showing the action of the non-conservative force and the Berry force combined was analysed in the theoretical work of Lü et al. [6]. In this work they consider a short chain of metal atoms suspended between metallic leads of the same metal (Au or Pt). They observe that a pair of nearly-degenerate vibration modes becomes coupled by the action of the current-induced forces, leading to negative damping of the atomic motion. In other words, the amplitude of the motion keeps increasing as a result of the energy that is pumped into the motion by the non-conservative force. In practice this will ultimately lead to a breakup of the wire.

This model system closely resembles the experimental configuration in experiments that were performed by Smit et al. [10]. In the discussion of their model calculations Lü et al. refer to these experiments and propose an alternative interpretation of the main peak in the experimental data in terms of their model of current-induced forces. Here we extend these experiments and argue that the main peak is still best described by regular thermally induced breaking as originally proposed by Smit et al. However, the pieces of the puzzle may fit better when considering the interpretation of an anomalous peak at low voltage that is observed in the experimental data. In the analysis by Smit et al. this peak was set aside as unexplained, and ignored in the analysis of the main results. We propose here that this unexplained peak may be the hallmark of the non-conservative forces acting at high current densities, and that it may provide the first experimental evidence for these effects. We extend the experimental data of [10] and focus our attention here on the analysis of the peak at low-bias voltage.

## Experimental

**Figure 1:**
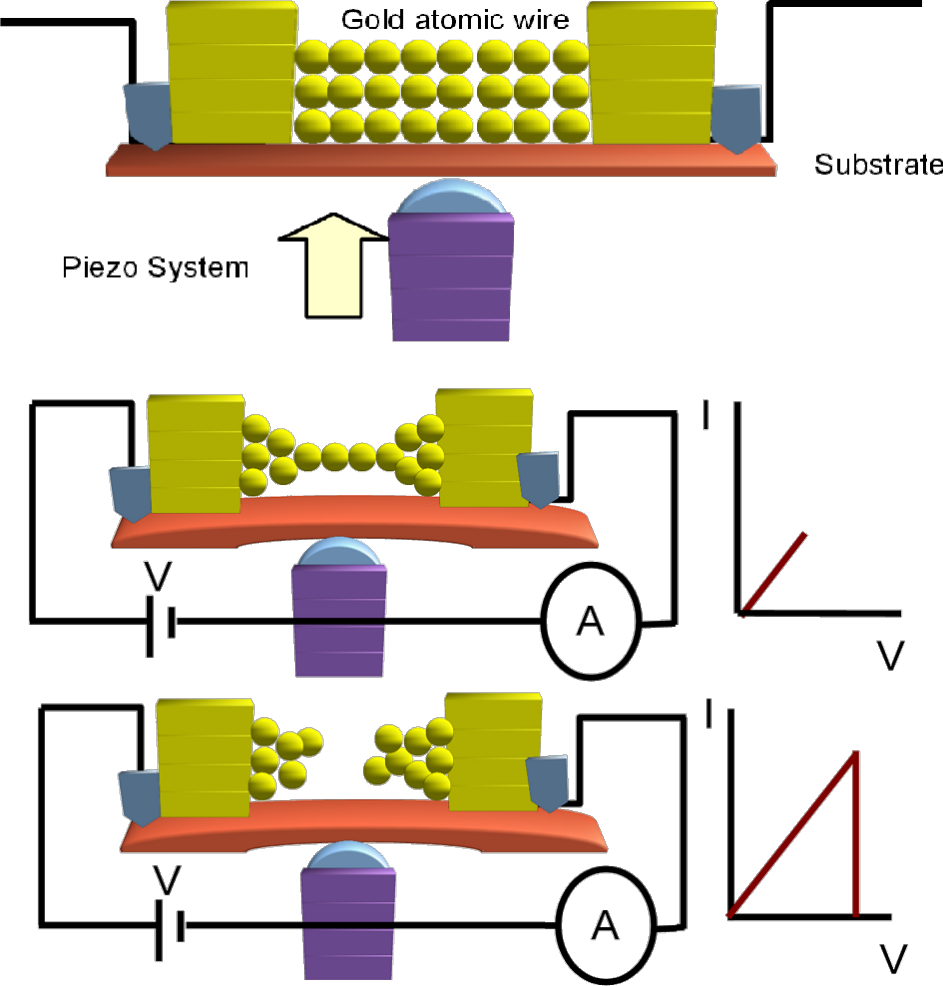
Schematic illustration of the MCBJ technique and the method of current-induced breaking. The top panel (not to scale) shows the initial configuration of a metal wire with a notch formed in the middle of the wire, and mounted in a three-point bending configuration. The middle panel illustrates the formation of an atomic chain after stretching the wire electrodes. The black lines represent the wires that connect the atomic sized structure to the bias voltage and to a current–voltage converter for recording the current. The small current–voltage diagram on the right emphasises that the currents and voltages remain low in this phase of the experiment. The bottom panel illustrates the current-induced breaking of the atomic chain.

For details of the experimental techniques we refer to Smit et al. [10]. Briefly, the experiment is based on the formation of chains of metal atoms by the mechanically controllable break junction (MCBJ) technique [11–12]. As schematically illustrated in Figure 1 we start from a macroscopic metallic wire that is mounted on a bending beam in a three-point bending configuration. A weak spot is introduced in the middle of the wire, which serves to initiate a break in the wire when the substrate is bent. The first break is only made once the wire is cold, at temperatures close to the boiling point of liquid helium, and under vacuum. This ensures that the fracture surfaces remain atomically clean, and the two wire ends can then be brought back into contact by relaxing the bending of the substrate. The MCBJ technique is a simple method for creating clean and stable single-atom contacts, and can in principle be applied to any metal.

The fine control of the bending and breaking is done by means of a piezo electric element (Figure 1). When gently breaking a small contact in this fashion at the last stages one observes a step-wise decrease in the conductance, which is due to the atom-by-atom break down of the contact. The conductance of the last atom bridging the gap depends on the type of metal used in the experiment [12–13]. This conductance is very close to the value of the quantum of conductance, *G*_0_ = 2*e*^2^/*h*, for simple monovalent metals, such as Au. When stretching the contact further, once a single atom bridge has been formed for most metals the contact breaks at this weakest spot that is formed by just one atom. Interestingly, for Au, Pt, and Ir it has been shown that one can continue stretching the contact while new atoms are folding in from the banks in order to self-assemble into an atomic chain, as illustrated in the middle panel of Figure 1 [14–16]. The sample wires used here were Pt and Au, have a diameter of 0.1 mm and a purity of 99.995%.

**Figure 2:**
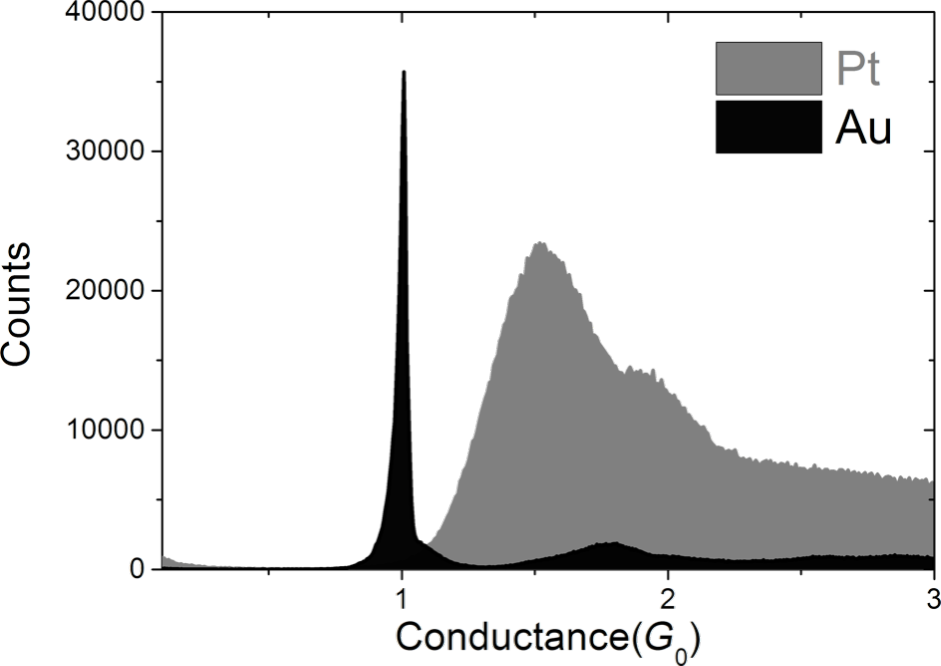
Histograms of conductance values for Au and Pt collected from 20000 individual breaking traces.

The goal of the experiment is to study the current-induced breaking of atomic chains of given length by a procedure that is schematically illustrated in the bottom panel of Figure 1. At the start of each experiment for a fresh sample wire the conductance properties of the atomic chain are characterized by recording a conductance histogram: While repeatedly moving the wire ends toward and away from each other a contact is repeatedly formed and broken. During these breaking cycles the conductance is recorded and the digitized values are collected into a histogram, see Figure 2. Clean metallic Au contacts are characterized by a high sharp peak at 1*G*_0_, while Pt has a much broader peak at 1.5*G*_0_. The difference in atomic-scale conductance between the two metals is due to the difference in numbers of conductance channels per atom and their associated transmission probabilities [12–13]. Au has just one conductance channel with nearly perfect transmission associated with the 6s orbital, while for Pt the 5d orbitals give rise to additional conductance channels with transmissions smaller than 1, due to limited matching to the wave functions of the bulk electrodes [17–18]. In both cases, Pt and Au, the peak in the conductance histogram is exceptionally strong, which can be understood from the fact that both metals develop atomic chains in the process of breaking, and these chains have conductance values in the range given by the peaks in Figure 2.

**Figure 3:**
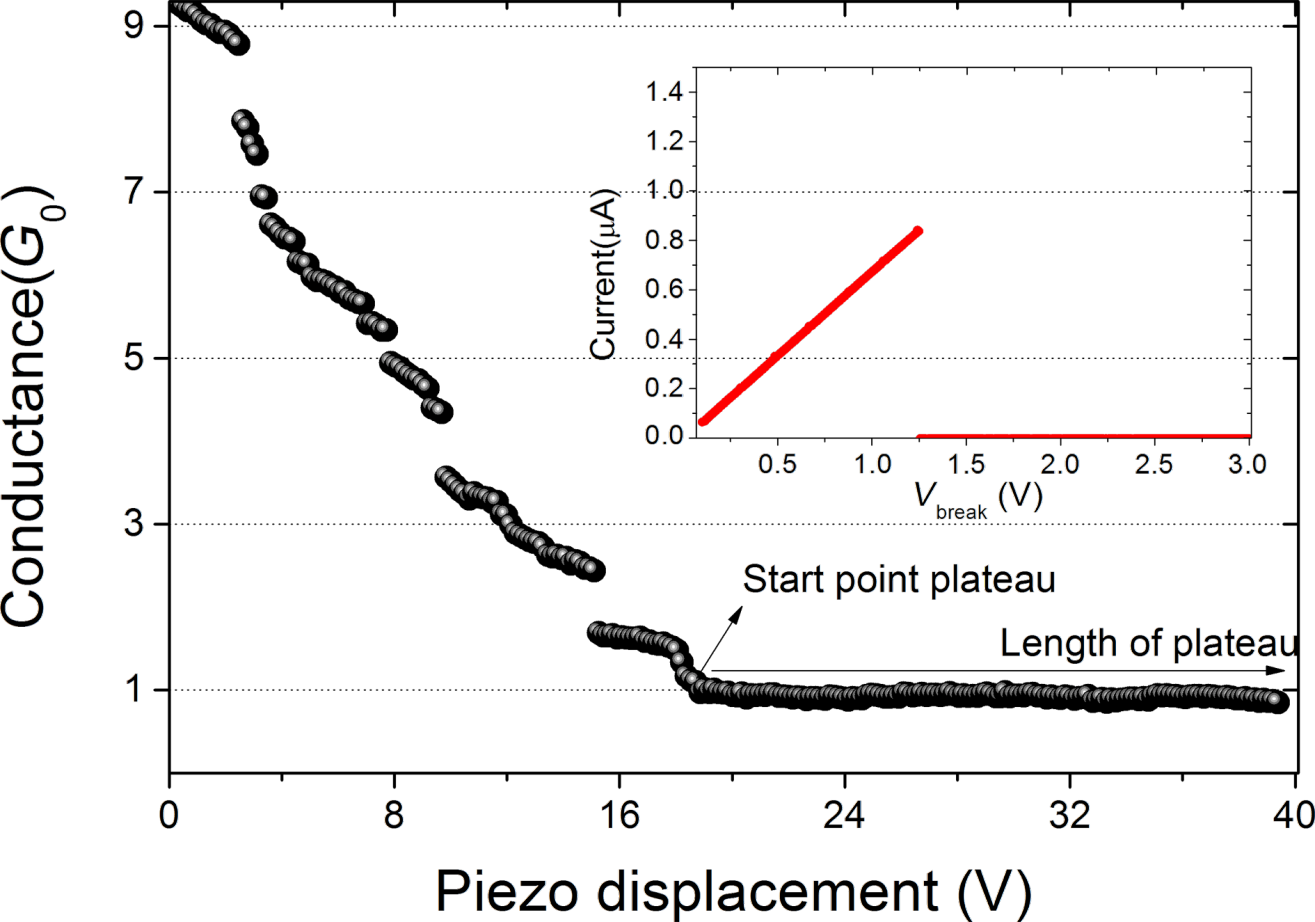
Example of a conductance breaking trace for Au. As soon as the conductance drops to the conductance of a single-atom junction, this point (arrow) is marked as the start of the formation of an atomic chain. Inset: Current vs bias voltage leading to breakup of the atomic chain at about 1.25 V.

An example of a long conductance plateau in a breaking phase of the cycle is shown for Au in Figure 3. The conductance is obtained as the ratio of the applied bias voltage (50 mV) to the current measured with the help of a current–voltage convertor. The conductance drops in steps as a result of atomic rearrangements taking place in the breaking process, and finally falls to a value below 1.2*G*_0_, which we take as the starting point of the building of an atomic chain. When continuing to increase the displacement of the wire ends with respect to each other the conductance remains at the same level of about 1*G*_0_, apart from small variations, as is expected for the buildup of an atomic chain. For the purpose of the present experiment we halt the displacement when a pre-set target length has been achieved, as measured from the starting point of the chain. In Figure 3 this is the distance between the arrow and the end of the graph at the right. In many cases, in particular when the target length is very long, a spontaneous break occurs before reaching the target. In those cases the curve is discarded and a new making-and-breaking cycle is started.

## Results and Discussion

Once we obtain a junction that reaches the target length without breaking, we halt the displacement of the wire ends and a routine is started that ramps the bias voltage from 50 mV up with a speed of 3 V/s. Well before the maximum voltage of 3 V is reached the junction breaks under the influence of the high current (inset in Figure 3). The bias voltage at which the break occurs, *V*_b_, is stored, together with other data characterizing the junction, including the atomic chain length (taken as the length of the last plateau), the break current, and the low-bias conductance. All these processes are controlled by software, and when a break voltage has been found, a new cycle is started automatically. In this way we have collected statistical information on the break voltage for atomic chains of Au and Pt, for a range of atomic chain lengths. The only difference in procedure between Au and Pt is the difference in conductance starting values for counting the length of the last plateau. We have used a start value of 2*G*_0_ for Pt and, although there is some ambiguity in the actual conductance at the start of chain formation for Pt, it has been shown that the choice does not influence the statistical data obtained [15].

**Figure 4:**
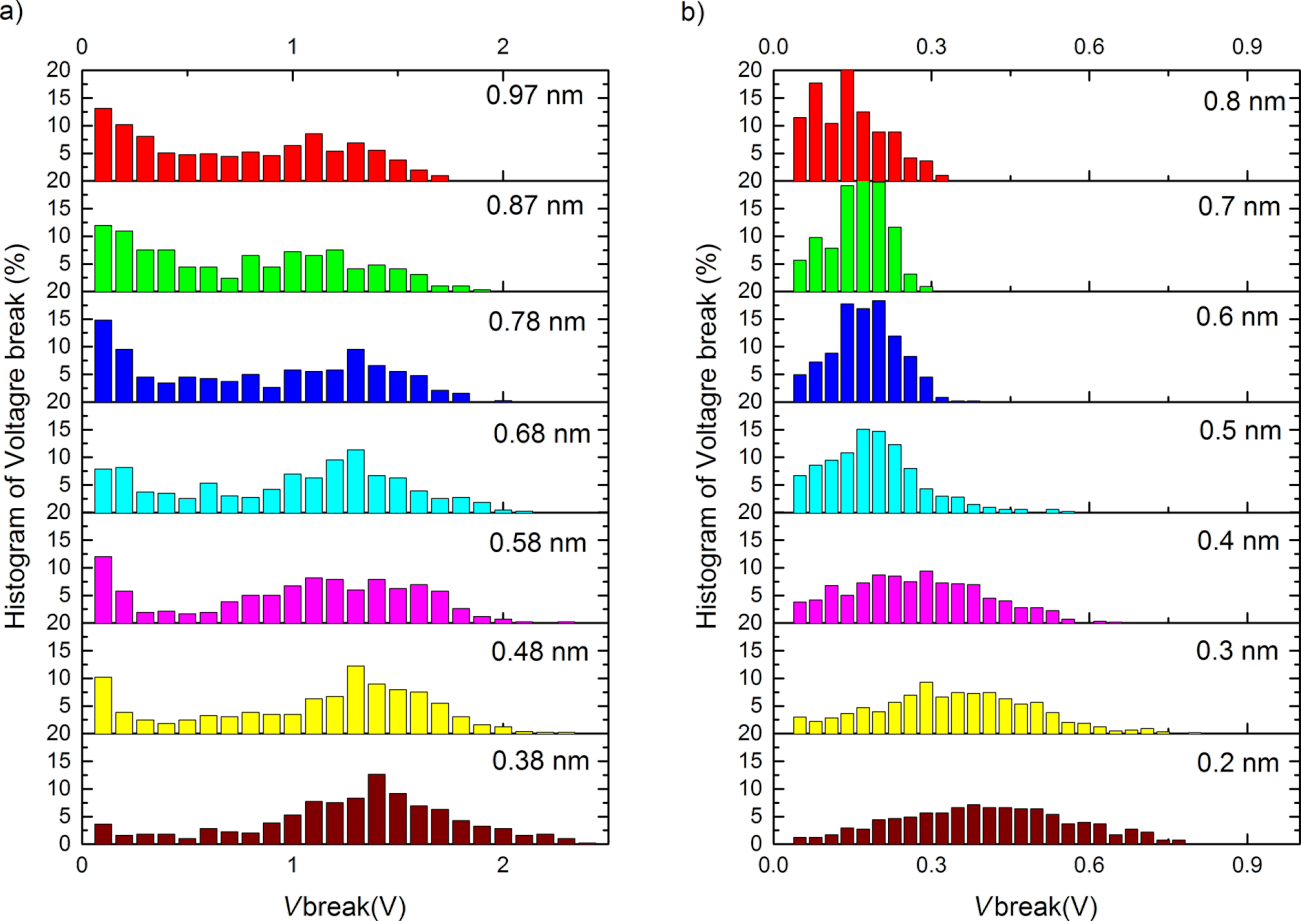
(a) Histogram of break voltage, *V*_b_, for Au atomic chains of lengths between 0.38 and 0.97 nm, as indicated in each of the panels. The results are shown as the percentage of all observed current-induced breaks. (b) Histogram of break voltage, *V*_b_, for Pt atomic chains of lengths between 0.2 and 0.8 nm.

The fraction of the total set of atomic chains, breaking at a given bias voltage is presented in Figure 4 in the form of histograms for each target length, for Au and Pt. The central observation for the purpose of this paper is the fact that the histogram for Au has two peaks: one at low break voltage, and a second broad peak that shifts from *V*_b_ = 1.4 V at a chain length of 0.38 nm down to *V*_b_ = 1.1 V at a length of 0.97 nm. Pt has only one broad peak shifting down from *V*_b_ = 0.42 V at a chain length of 0.2 nm to *V*_b_ = 0.15 V at 0.8 nm. The position of the lower peak for Au remains fixed at the lowest voltage values, but grows in intensity and width with increasing length of the atomic chains.

The results reported above confirm the data reported earlier by Smit et al. [10]. The structure of the break-voltage distribution for Pt, and the two-peak distribution for Au agree with those reported by Smit et al. The broad peaks at finite bias for Au and Pt are found at slightly higher values, except for the lowest two lengths for Pt that lie about 30% higher than in the work by Smit et al. The difference is possibly related to the fact that the initial preparation of a chain of a given length is done here at a higher bias voltage (50 mV) compared to that used by Smit et al. (10 mV). By using a higher bias at preparation we effectively eliminate chain configurations that are unstable at low-bias voltages, which affects the shape of the distribution. The broad peaks at finite break voltage for Au and Pt have been attributed to a mechanism of thermal breakup of the atomic chains in [10], and it was demonstrated that a simple model involving Joule heating produces a reasonable description. The model assumes the ensemble of breaking events can be described by thermal activation over a distribution of barrier heights. Dissipation raises the effective temperature above the bath temperature *T* according to 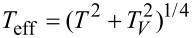 [19], with being *T_V_* determined by the applied bias voltage as 
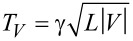
 [20], where γ = 60 K·V*^−^*^1/2^·nm*^−^*^1/2^ and *L* is the length of the chain. The parameters influencing the barrier height were taken from experiments, but the tensile stress will vary depending on the atomic arrangements at the end points of the chain and is not easily obtained from the experimental data. Therefore, this was assumed to be the main factor giving rise to variations in the barrier. The tensile force was assumed to be given by a normal distribution centred at 0.95 nN, and a width of 0.3 nN. A simulation of breaking events based on these assumptions reproduces the main characteristics of the second peak in the experimental distribution, including its shape and its length dependence. As mentioned above, in the analysis of [10] the lower peak in the break-voltage distribution for Au was set aside, and has remained unexplained.

**Figure 5:**
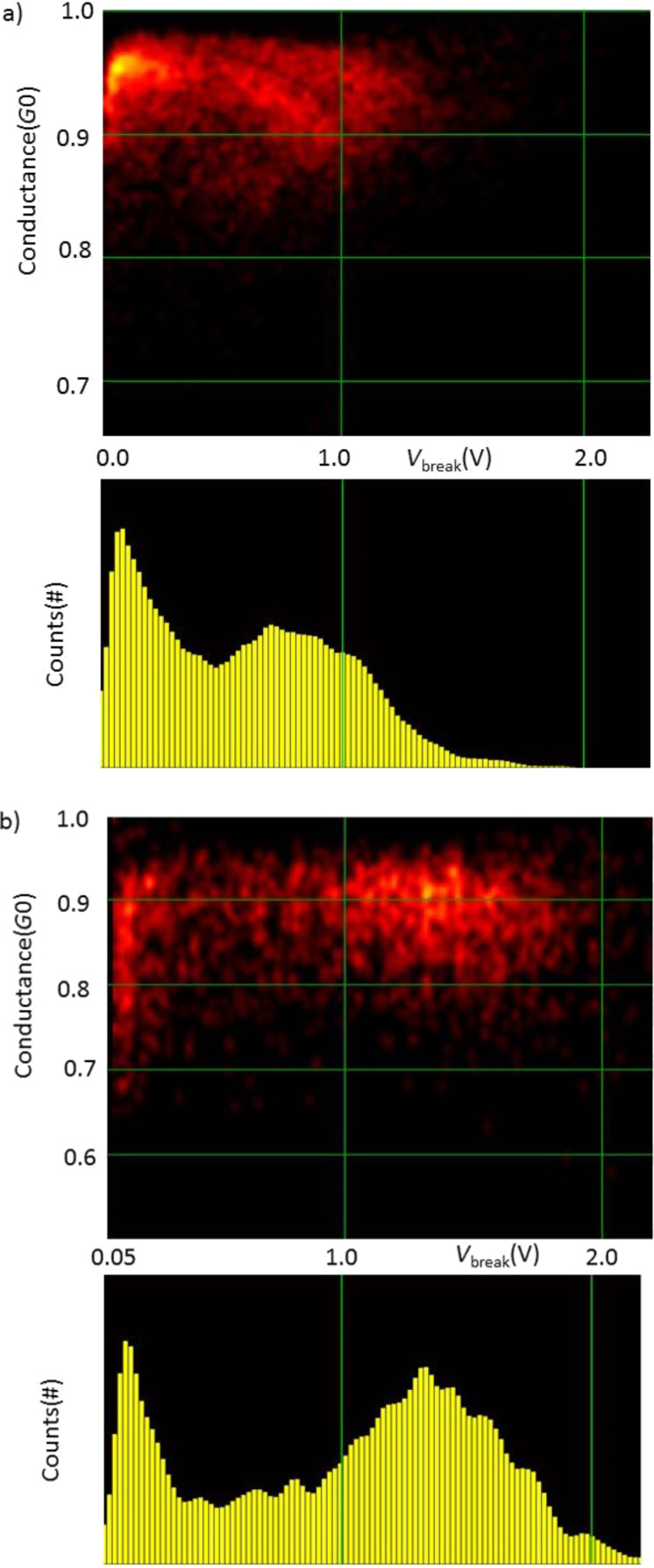
(a) Density plot (top panel) and histogram (lower panel) collecting all breaking voltages for all chain lengths for Au at 10 mV preparation voltage, as obtained from the data in [10]. (b) Density plot (top panel) and histogram (lower panel) collecting all breaking voltages for all chain lengths for Au at 50 mV preparation voltage.

**Figure 6:**
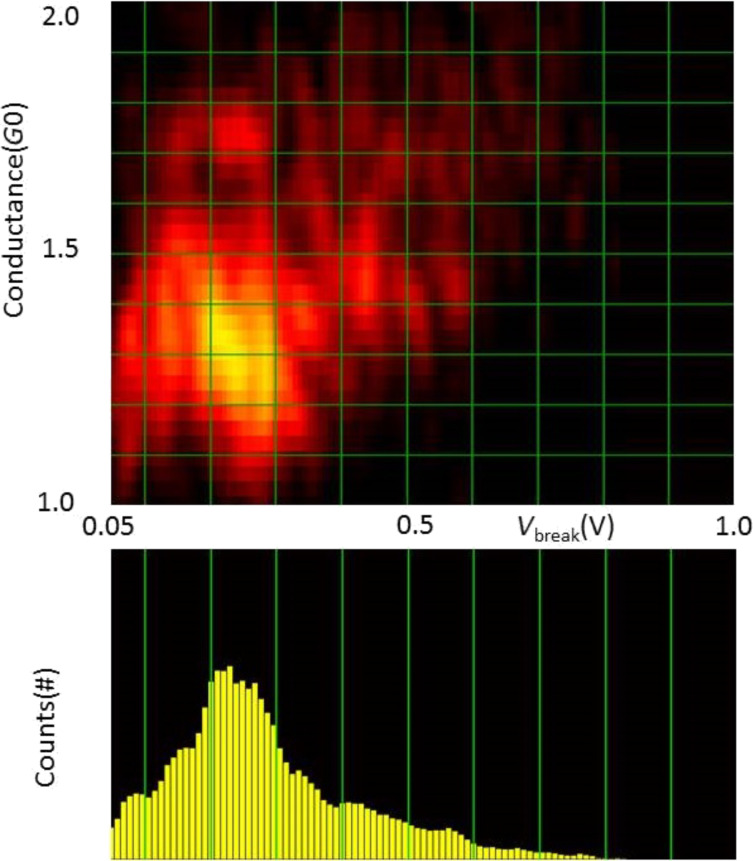
Density plot (top panel) and histogram (lower panel) collecting all breaking voltages for all chain lengths for Pt at 50 mV preparation voltage

Figure 5 shows density plots and histograms for all the data collected for all chain lengths for Au, with the break voltage given along the abscissa and the low-bias conductance along the ordinate. The data collected here for a preparation voltage of 50 mV, given in Figure 5b, is compared to the data set from [10] given in Figure 5a. The data clearly show that there are two disjunct regions of maximum density in the plots, and the separation between the two regions is enhanced by the higher preparation voltage (panel b). For Pt, Figure 6 only displays a single region of high density. We will focus our discussion now on the nature of the region of high density at the left side in the plots for Au.

The breaking of the atomic chains at low bias is remarkable for at least two reasons. First, we verified that the atomic chains survive for very long times (hours) when the displacement of the electrodes is kept fixed and also the bias voltage is held at 50 mV, or 10 mV depending on the experiment. As soon as we ramp up the bias a large fraction of the chains break spontaneously, even below 100 mV. For higher bias voltages the fraction of chains breaking decreases. Second, after seeing the probability for breaking decrease, it rises again towards a second peak. These observations are difficult to explain assuming a homogeneous population of chain configurations. If every newly formed chain is subject to the same mechanisms of current-induced breaking once a process is activated, more current should lead to a smaller probability to survive to that level of current.

For a given mechanism of current-induced breaking there may be a threshold, or optimum, value for the mechanism. Indeed, this is what was considered by Smit et al. for the second region for Au at higher bias, where a certain level of Joule heating is needed for breaking to have a high probability. After this optimum breaking voltage the probability of breaking decreases, not because it is less likely for a chain to break at higher bias. On the contrary, a higher bias should always enhance the probability of breaking. However, because less of the chain population survives to such high bias there are fewer left to break at the higher voltage, explaining the decaying rate of breaking observed.

Through this reasoning we conclude that a decaying rate of breaking indicates a decaying survival of the population. This implies that the first region for Au must be associated with a subset of chain configurations that possess a property that sets it apart from the other subset that is characterized by regular breaking due to Joule heating. Each time the contact is stretched to form an atomic chain the atoms arrange themselves in different ways. Some of these arrangements make the chains susceptible to a mechanism that leads to breaking at low bias, while the other arrangements are insensitive to this mechanism.

In principle the two subsets of chain configurations can differ in many ways, e.g., by the way the atoms are arranged at the ends of the chain, or by the states of strain. The difficulty that we meet is that such variations are expected to give rise to a somewhat continuous range of possibilities, and this range has already been included in the model describing the second peak in the distribution for Au. One would need a property that sets a fraction of chain configurations apart, not just quantitatively, but qualitatively.

The non-conservative force mechanism discussed in [4–7] provides an appealing candidate for such a mechanism. The mechanism requires the presence of two nearly degenerate vibration modes with strong coupling to the current. When these conditions are met the combined action of pumping of energy into the modes by the non-conservative force and the periodic motion in phase space imposed by the Berry force lead to a growing amplitude of the combined motion of the two modes. As the amplitude continues to grow at a given moment the chain will become unstable and break.

Thus, we propose the non-conservative forces as a tentative explanation for the anomalous region of low-bias chain breaking that is observed for Au. In the population of atomic chain configurations those that have a set of suitable nearly-degenerate vibration modes fall into the subset that belongs to the low-bias region of current breaking. The other atomic chain configurations, which may have a less symmetric arrangement of the atoms at the connections to the banks, will only break by regular thermal activation due to Joule heating. The threshold of about 0.2 V for a runaway mode for Au obtained in the model calculation of Lü et al. [6] agrees with the range of the low-bias breaking voltages observed in our experiments.

The absence of a disjunct low-bias voltage region for Pt may be explained by the fact that the runaway modes for Pt in the calculation appear at higher bias, 0.4 V, while at the same time the thermal breaking takes place at lower bias as compared to Au. This would indicate that the bias regions for both mechanisms for Pt overlap.

The current-induced breaking mechanism of Lü et al. [6] has a sharp threshold voltage, which we do not observe here. This can be understood by the fact that the experiment shows the result of a population of chain configurations with widely different atomic arrangements. For each of these chain configurations the threshold may be different. Since we cannot discern a low-bias maximum for Au at finite bias, we must assume that a fraction of the chain configurations has a threshold below the initial bias voltage used during the preparation of the chains. Since these chains will be lost during the chain preparation process they are not included in the statistics. The qualitative agreement between the distributions obtained for 10 and 50 mV preparation voltage indicates that breaking below 50 mV becomes less probable.

From the density plots for Au in Figure 5 we further note a trend in the region of low-bias breaking towards lower conductance the lower the break voltage of the chains. This suggests that the transmission for these anomalous chain configurations is lower, which gives another indication that the configurations are exceptional. The density plot for Pt, Figure 5, also shows a slight trend, on a much wider scale, of increasing conductance for higher break voltage. In this case this is likely due to lumping of the short and long chain data in one plot. Pt has several conductance channels with transmissions that depend on the structure, where short Pt atomic chains have a higher conductance [21].

A full comparison of theory and experiment will require further work on both fronts. Experimentally, progress would require methods to determine the atomic arrangement of atomic chains, or the investigation of other atomic scale systems that permit a higher degree of control. On the side of theory the effects of phonon decay have not been included. Qualitative estimates of the effect of phonon decay suggest that they can have a profound influence on the final size of the non-conservative force and the runaway modes [6].
